# Predictors of stethoscope contamination following a standardized physical exam

**DOI:** 10.1186/1753-6561-5-S6-P304

**Published:** 2011-06-29

**Authors:** A Schneider, C Tschopp, Y Longtin, G Renzi, A Gayet-Ageron, J Schrenzel, D Pittet

**Affiliations:** 1 Geneva University Hospital, Geneva, Switzerland

## Introduction / objectives

The relative contribution of stethoscopes in microbial cross-transmission in comparison to the examiners’ hands has not been well described. The aim of this study is to compare stethoscope versus hand contamination following a physical exam and identify predictors of stethoscope contamination.

## Methods

Following a standardized physical exam using sterile gloves and a sterile stethoscope, bacterial contamination of the following regions were assessed using contact plates: stethoscope diaphragm, stethoscope tube, fingertips, thenar region, hypothenar region and back of physician’s dominant hand. Total aerobic colony count (ACC) were determined on digital photographs using a counting tool.

## Results

A total of 56 patients (62% males; median age, 66) were recruited. Median (IQR) contamination (in ACC/25cm2) of examiner’s dominant hand and stethoscope were as follows: fingertips: 835 (IQR, 332-1638), stethoscope diaphragm: 173 (IQR, 36-535), stethoscope tube: 116 (IQR, 34-321), hypothenar region: 16 (IQR, 8-59), thenar region: 15 (IQR, 4-71) and dorsum of hand: 3 (IQR, 1-16). The stethoscope diaphragm and tube were significantly more contaminated than the thenar or hypothenar regions (Wilcoxon ranksum test: p<0.001).There was no difference between the level of tube and diaphragm contamination. Diaphragm contamination was strongly associated with the patient’s level of skin contamination (p<0.001), the patient’s BMI (p=0.01) and the degree of humidity of the patient’s skin (p<0.001).

## Conclusion

Our results suggest that stethoscopes’ diaphragm and tube are significantly contaminated following a physical exam and identify predictors of heavy contamination. These findings suggest the need to decontaminate stethoscopes following each use.

## Disclosure of interest

None declared.

